# Biofilm-Forming Potential of Ocular Fluid *Staphylococcus aureus* and *Staphylococcus epidermidis* on Ex Vivo Human Corneas from Attachment to Dispersal Phase

**DOI:** 10.3390/microorganisms9061124

**Published:** 2021-05-22

**Authors:** Ranjith Konduri, Chinthala Reddy Saiabhilash, Sisinthy Shivaji

**Affiliations:** Jhaveri Microbiology Centre, Prof. Brien Holden Eye Research Centre, Kallam Anji Reddy Campus, L. V. Prasad Eye Institute, Hyderabad 500034, Telangana, India; kranjith@lvpei.org (R.K.); abhilashreddy495@gmail.com (C.R.S.)

**Keywords:** biofilm, ocular fluid, antimicrobial resistance, eye, *S. aureus*, *S. epidermidis*

## Abstract

The biofilm-forming potential of *Staphylococcus aureus* and *Staphylococcus epidermidis*, isolated from patients with Endophthalmitis, was monitored using glass cover slips and cadaveric corneas as substrata. Both the ocular fluid isolates exhibited biofilm-forming potential by the Congo red agar, Crystal violet and 2,3-bis (2-methoxy-4-nitro-5-sulfophenyl)-5-(phenylamino) carbonyl-2H-tetra-zolium hydroxide (XTT) methods. Confocal microscopy demonstrated that the thickness of the biofilm increased from 4–120 h of biofilm formation. Scanning electron microscopic studies indicated that the biofilms grown on cover slips and ex vivo corneas of both the isolates go through an adhesion phase at 4 h followed by multilayer clumping of cells with intercellular connections and copious amounts of extracellular polymeric substance. Clumps subsequently formed columns and eventually single cells were visible indicative of dispersal phase. Biofilm formation was more rapid when the cornea was used as a substratum. In the biofilms grown on corneas, clumping of cells, formation of 3D structures and final appearance of single cells indicative of dispersal phase occurred by 48 h compared to 96–120 h when biofilms were grown on cover slips. In the biofilm phase, both were several-fold more resistant to antibiotics compared to planktonic cells. This is the first study on biofilm forming potential of ocular fluid *S. aureus* and *S. epidermidis* on cadaveric cornea, from attachment to dispersal phase of biofilm formation.

## 1. Introduction

The eye has a number of defense mechanisms including several components in the tears (lysozyme, immunoglobulins, lactoferrin, lipocalin, β-lysin, etc.) which act as the first line of defense against bacterial infection [1,2,3]. Blinking the eyelids also plays an important role in the spread of tears across the ocular surface and thus acts as a barrier to the microbial-colonization of the ocular surface [4]. The cornea also has an immune surveillance system [5,6] comprised of innate defenses contributed by numerous cellular (corneal epithelial cells, corneal nerves, keratocytes, polymorphonuclear cells, neutrophils, eosinophils, macrophages, NK cells, Langerhans cells, etc.) and molecular elements (components of complement, interferons, interleukins, etc.) to eliminate pathogens [5,6,7]. Despite these defense mechanisms, many microorganisms do survive on the ocular surface and recent studies either based on 16S ribosomal RNA (rRNA) gene amplification, cloning and sequencing or by using NGS (next-generation sequencing) based on 16S rRNA gene amplification and analysis (16S rRNA meta-barcoding) revealed a greater degree of diversity and abundance in the bacterial microbiome of the ocular surface [8,9,10,11,12,13]. Keilty [14] was the first to cultivate hemolytic *Staphylococcus* from the conjunctival swabs of normal subjects. Subsequently, it was observed that several Gram-positive bacteria, including coagulase-negative staphylococci (CoNS) and other staphylococcal species such as *Staphylococcus aureus*, *S. epidermidis*, and a species of Streptococcus, *S. viridans* cause acute postoperative endophthalmitis [15,16,17,18,19] and other ocular infections such as endophthalmitis, keratitis, Scleral buckle infection, Lacrimal system infections, Periorbital infections, etc. [20,21,22,23]. *Staphylococcus aureus* and *S. epidermidis* are also the leading cause of infection of ocular implants such as intra-ocular lenses, Scleral buckles, Conjunctival plug, Lacrimal intubation devices, etc. [21,24,25,26].

The virulence of the above *Staphylococcus* spp. predominantly found on the ocular surface has been attributed to their ability to form a biofilm which confers antimicrobial resistance [20,26,27,28]. A biofilm is a community of microbes sequestered in a self-secreted matrix, the extracellular polymeric substance (EPS) [29,30,31]. A biofilm, in addition to having the microbial cells and EPS, also has a defined unique architecture [29,30,32,33,34] defined by some structural attributes similar to water channels [35], the thickness (varying from monolayer of cells to three-dimensional structures similar to columns) and the presence of voids. Voids are normally detected by time lapse monitoring of the biofilm after every 15 min. After the biofilm has matured and reached a sufficient size, cells detach creating voids. However, with time additional growth occurs in the voids left by detached cells [36,37,38]. In the biofilm-phase, the cells are protected from the killing effect of an anti-microbial agent and the biofilm also confers protection against the hostile environment and host defense mechanisms [39]. A characteristic feature of the bacteria involved in biofilm formation is its transition from a planktonic phase to a sedentary life style on a surface [40]. This transition occurs in four distinct stages: adhesion (when planktonic cells adhere to s substratum), microcolony formation (when bacteria proliferate and get organised into multi-layered cellular structures), maturation (when the biofilm appears as vertical columns or mushroom-like 3 dimensional assemblies enclosed in EPS) and, finally, the dispersion phase. In the dispersal phase, individual cells and/or multicellular aggregates are dispersed from the mature biofilms to seed new biofilms [20,41,42]. EPS, the non-viable component of a biofilm, is a gelatinous material comprising of proteins, polysaccharides, nucleic acids, lipids, dead bacterial cells, and other polymeric substances hydrated to 85–95% water [29,30] and has several attributes. EPS is of two types: soluble EPS (weakly bound with cells) and bound EPS (closely bound with cells) which could be either loosely bound EPS (LB-EPS) and tightly bound EPS (TB-EPS) [43]. EPS maintains the structural integrity of the biofilm, anchors the biofilm to a substratum [44], facilitates cell to cell communication and the viability of cells by modulating substrate absorption, oxygen diffusion and transport of molecules within the biofilm [45,46]. Biofilms can account for more than 80 percent of microbial infections. Thus, it is important to study biofilm biology because it impacts both animal and human health [29,30,31] by conferring protection to bacteria from the lethal effects of antibiotics, disinfectants and host immune response. Studies directed towards in vitro biofilm formation on several different types of substrates including abiotic substrates such as microtiter plate systems, flow cells, the constant depth film fermenter, annular reactors and the perfused biofilm fermenter [47,48] and biotic substrates such as body tissues, mammary alveolar cells or the skin of fruits [49,50] would be very relevant to the understanding of the biology of biofilms.

*Staphylococcus aureus* and *S. epidermidis* are also commonly found infecting the anophthalmic cavity of ocular prosthesis users [51,52]. Some or all the previous studies on biofilm formation in *S. aureus* and *S. epidermidis* used non-clinical strains [53], whereas, in this study, both *S. aureus* and *S. epidermidis* were of clinical origin, isolated from patients with infectious Endophthalmitis. Biofilm formation was monitored by Congo red (CR) method as in the previous studies [54]; but, for monitoring the temporal dynamics of biofilm formation, we used the Crystal violet (CV) method [54] and also the XTT method [55]. Temporal dynamics of the biofilm were rarely monitored. In this study, Confocal laser scanning microscopy (CLSM) was used to monitor the temporal changes in the thickness of the biofilm [56,57]. Additionally, scanning electron microscopy (SEM) was used to visualize the biofilm from attachment to dispersal phase as in a few earlier studies [53,54,58]. A unique feature of this study is that in addition to using cover slips, human donor corneas were also used as a substratum for monitoring biofilm formation. This approach of using cadaveric cornea as a substratum is important since bacteria colonize the cornea, allow prolonged survival of microorganisms and are the cause of active inflammation and infection [59]. Further, antibiotic susceptibility was monitored both in the planktonic and biofilm phases. The results confirmed, based on qualitative (Congo red agar (CR) and Scanning Electron Micrscopy (SEM)) and quantitative methods (Crystal Violet Method (CV), [2,3-bis (2-methoxy-4-nitro-5-sulfophenyl)-5-(phenylamino) carbonyl-2H-tetra-zolium hydroxide)] (XTT) and Confocal Laser Scanning Microscopy (CLSM)), that the two ocular isolates possess the potential to form biofilm. More importantly, SEM analysis of the biofilms indicated that ocular fluid *S. aureus* and *S. epidermidis* produced biofilms both on a synthetic substratum and on cadaveric cornea. However, biofilm formation on the cadaveric cornea was more rapid and cornea was probably the preferred substratum. This is the first study on biofilm forming potential of ocular fluid *S. aureus* and *S. epidermidis* on cadaveric cornea, from attachment to the dispersal phase of biofilm formation.

## 2. Materials and Methods

### 2.1. Bacterial Cultures and Characterisation

In the present study, two vitreous samples from patients with Endophthalmitis were received from the L V Prasad Eye Institute, Hyderabad, India, and cultured on 5% sheep blood agar medium plates [23] and two single colonies were purified by repeated streaking and subjected to basic microbiological tests. Both the isolates were Gram positive, coccoid, occurred in groups and were positive for catalase. Isolate L-1058-2019 (2) produced pink color colonies on MSA agar and white opaque color colonies on non-hemolytic blood agar, and was negative for coagulase and oxidation-fermentation test, suggestive of *Staphylococcus epidermidis.* In contrast, isolate L-1054-2019 (2) produces yellow color colonies on MSA agar and cream white opaque color colonies on β-hemolytic on blood agar and is positive for coagulase and oxidation-fermentation test suggestive of *Staphylococcus aureus.* The identity of the two isolates as *Staphylococcus aureus* and *S. epidermidis* was also confirmed using Vitek 2 Compact System (BioMérieux, Marcy l’Etoile, France), an automated system for the identification of bacterial isolates up to species level. Vitek 2 has been reported to identify 95% of *Staphylococcal* isolates correctly [60]. The two isolates were preserved in tryptone soya broth (TSB) [61] with 30% glycerol at −80 °C. All the preserved isolates were revived on 5% sheep blood agar media plates and incubated overnight at 37 °C. Henceforth, the above two isolates would be referred to as ocular fluid isolates.

### 2.2. Determination of Biofilm Formation by Various Methods

*Staphylococcus aureus* and *S. epidermidis* were tested for their ability to form biofilm by congo red agar (CRA), crystal violet (CV) and XTT [2,3-bis (2-methoxy-4-nitro-5-sulfophenyl)-5-(phenylamino) carbonyl-2H-tetra-zolium hydroxide)] methods as described earlier [62,63].

#### 2.2.1. Congo Red Agar Method

The CRA method is a qualitative assay for monitoring the potential of a microorganism to form a biofilm when grown on a solid CRA plate [64]. Congo red is known to bind to amyloid-like proteins which are a component of the extra-cellular polymeric substance of the biofilm [65,66]. In this method a single colony of *S. aureus* and *S. epidermidis* were cultured on a CRA plate [containing (37 g/L) Brain Heart Infusion (BHI), (50 g/L) sucrose, (10 g/L) agar and (8 g/L) Congo Red indicator (Himedia, Secunderabad, India)] at 37 °C for 24 h. Black colored colonies were indicative of biofilm positive isolates [64,65,66].

#### 2.2.2. Crystal Violetmethod

The CV method is a quantitative method which monitors biofilm mass. CV is a positively charged molecule which binds to negatively charged bacteria and polysaccharides of EPS which could be quantitated [67,68,69]. In this method an overnight culture was diluted 10,000 times (*v*/*v*) and then 100 µL of this suspension was added to a 96 well polystyrene plate (Nunclon™, Thermo scientific, Roskilde, Denmark) containing 100 μL of BHI medium. Cultures were incubated at 37 °C for 4, 24, 48, 72, 96 and 120 h, after which the broth was decanted, the wells washed twice using 200 µL of phosphate-buffered saline, pH 7.4 (1X PBS contains 137 mM NaCl, 2.7 mM KCl, 10 mM Na_2_HPO_4_ and 1.8 mM KH_2_PO_4_) (PBS) (Sigma Chemical Co., St. Louis, MO, USA), plates air dried at room temperature (RT) and bacterial cells that had adhered to the wells were stained using 0.1% CV (Sigma Chemical Co., St. Louis, MO, USA). Excess crystal violet was discarded and each well was washed twice with 200 µL of PBS and dried at RT. CV associated with the bacteria was extracted with 200 μL of absolute ethanol and quantified using a Spectrophotometer [SpectraMax M3, with a cuvette adaptor (Molecular Devices, San Jose, CA, USA)] set at 595 nm [67,68,69]. Wells without cells served as the control (OD was < 0.1 at 595 nm) and the OD value was deducted from the biofilm positive (OD > 0.3 at 595 nm) and biofilm negative strains (OD < 0.3 at 595 nm) [62,63]. The experiment was performed with three replicates.

#### 2.2.3. [2,3-Bis (2-methoxy-4-nitro-5-sulfophenyl)-5-(phenylamino) carbonyl-2H-tetra-zolium hydroxide)] Method (XTT)

XTT was also used for the detection of biofilm [63,70]. The assay is based on the cleavage of the tetrazolium salt XTT to water-soluble formazan (orange color) due to the metabolic activity of the organism. Formazan formed is quantitated using a scanning multi-well spectrophotometer. The measured absorbance directly correlates to the number of viable cells. In this method cultures were diluted 10,000 times and incubated as in the CV method. Subsequently, media was decanted, each well washed twice using 200 µL of autoclaved milliQ water and allowed to air dry for 30 m. Freshly prepared 200 μL of XTT solution [147 µL of PBS and 50.5 µL of XTT (1 mg/mL, Sigma Chemical Co., St. Louis, MO, USA) and 2.5 µL of Menadione (0.4 mM, Sigma Chemical Co., St. Louis, MO, USA)] was added to each well and incubated in the dark at 37 °C for 3 h. From each well, 100 μL was then transferred to a new 96 well plate and biofilm formation was quantified using a was quantified at 490 nm using a SpectraMax M3, microplate reader (Molecular Devices, CA, USA). A well without the inoculums served as a blank and *E. coli* ATCC 25922 and *S. aureus* ATCC 25923 were used as negative and positive control, respectively, for biofilm production. Experiment was performed with three replicates.

### 2.3. Monitoring the Thickness of the Biofilm and Visualisation of Extracellular Polymeric Substance (EPS) by Confocal Laser Scanning Microscopy

EPS and thickness of the biofilm (on cover slips) was monitored by CLSM using dual staining [63]. An overnight culture was diluted as in the CV and XTT methods and the bacterial suspension was added to a glass cover slip (Blue star, Mumbai, India) placed in the well of a 12 well polystyrene plate (Nunclon™, Thermo Scientific, Roskilde, Denmark) and incubated at 37 °C for 4, 24, 48, 72, 96, and 120 h. After the incubation period, the cover slips were washed with autoclaved distilled water and fixed with 250 µL of formaldehyde (4%) for 3 h. Fixed biofilms were then washed twice as above and stained for 30 min with 200 μL of 1.67 μM Syto^®^9 (Invitrogen, Carlsbad, CA, USA), a nuclear fluorescent dye, that stains DNA of viable cells and emits green color. After staining with Syto 9, biofilms were stained in the dark with 0.025% Calcofluor white M2R (Sigma Chemical Co., St. Louis, MO, USA) for 30 min. This dye binds to β-linked polysaccharides and fluoresces under long-wave UV light and biofilm could be visualized (blue) using confocal microscopy. Calcofluor white was excited at 363-nm using a 455/30 band-pass filter [71] and emits blue color. The thickness of the biofilm at each time point was measured across the entire biofilm and the values are reported as (Z axis, Average ± standard deviation in µm). Calcofluor white has been used to study exopolysaccharides (EPSs) involved in biofilm formation in a variety of organisms [72].

### 2.4. Visualisation of Biofilm on Cover Slips by Scanning Electron Microscopy

Cultures were processed for biofilm formation as above on glass cover slips (Blue star, Mumbai, India) and were then transferred to a new 12 well polysytrene plate (Nunclon™, Thermo scientific, Roskilde, Denmark), washed thrice with autoclaved distilled water and fixed for 3 h with 250 µL of glutaraldehyde (2.5%). After fixation, the glass cover slip was washed thrice with autoclaved distilled water, dehydrated for 20 m through graded ethanol (10, 25, 50, 70, 90 and 100%) and finally air dried overnight. Biofilms on the cover slips were sputtered with gold for 60 s using a High Vacuum Evaporator (SC7620 PALARON Sputter Coater, Quorum Technologies Ltd., East Sussex, UK) and visualized using a scanning electron microcope (SEM) (Carl Zeiss-Model EVO 18, Carl Zeiss, Germany). The Voltage used for acquiring the SEM images ranged between 5–20 kV.

### 2.5. Visualisation of Biofilm on Human Cadaveric Cornea by Scanning Electron Microscopy

Human cadaveric cornea, which do not meet the stringent quality required for transplantation were obtained from The Ramayamma International Eye Bank (RIEB), LVPEI, Hyderabad, India. All corneas were obtained following procedures approved by the institutional review board for the protection of human subjects. Corneas were received in MK medium containing gentamicin [73]. Therefore, they were thoroughly washed with PBS and biofilm formation was set up as described earlier [74]. The cadaveric corneo-scleral button (cornea + 2 mm of peripheral sclera) was placed on a 35 mm Petri-dish with the endothelial layer facing up. In this orientation, the cornea appears as a shallow cup. Into this cup, 500 μL of a semisolid Dulbecco’s modified Eagle’s medium (DMEM) (© 2021 Merck KGaA, Darmstadt, Germany) with agarose (0.5% *w*/*v*) was transferred and allowed to solidify. The corneas were then inverted so that the epithelial side was now facing upward. The cornea was then immersed in an antibiotic free DMEM culture medium containing 10% fetal calf serum, 5 μg/mL insulin and 10 ng/mL epidermal growth factor and incubated for 24 h at 37 °C in a 5% CO_2_ incubator, to remove the residual antibiotics. Corneas from the antibiotic free DMEM medium were washed with PBS and a sterile steel scalpel was used to create three vertical and horizontal cuts [74]. Subsequently, the bacterial inoculum from an overnight culture grown in BHI media was diluted 10,000 times with BHI broth and centrifuged at 12,000 rpm (Eppendorf USA, Framingham, MA, USA, model no: 5430) for 5 min at room temperature (25 °C) and the pellet washed with 200 μL of autoclaved distilled water and centrifuged. The final pellet was suspended in 100 μL of DMEM (without fetal calf serum and antibiotics) and was gently transferred onto the surface of the corneas and incubated for 4, 24, 48, 72, 96, 120 h at 37 °C in a CO_2_ incubator (5% CO_2_ in air). After the incubation period, the cornea were processed for SEM to visualize biofilms on the cornea.

### 2.6. Antibiotic Susceptibility in Planktonic and Biofilm Phase

Several antibiotics were evaluated for their antimicrobial activity as per Clinical and Laboratory Standards Institute guidelines [75]. For this purpose, the overnight bacterial suspension in BHI was diluted 10,000 times and 100 μL of the suspension was added to each well of the 96 well polystyrene plate (Nunclon™, Thermo scientific, Roskilde, Denmark) containing 100 μL of an antibiotic of a known concentration [62,63]. Minimum inhibitory concentration (MIC) for each antibiotic was determined, as outlined in the CLSI-M07-A10 guidelines (CLSI, 2012).

For monitoring the inhibitory effects of the antibiotics, in the biofilm phase, cultures were allowed to form biofilms in the 96 well plate for 96 h, washed twice with PBS to remove planktonic bacteria and then known concentrations of the antibiotics were added and further incubated for 24 h. After incubation, the wells were gently washed using 200 μL of PBS to remove the free cells and the plates were then processed for monitoring the effect of the compound on the biofilm by the XTT method [62,63]. Inoculums without the addition of the compound served as a negative control. All experiments were performed in triplicate.

## 3. Results

### 3.1. Biofilm Formation in Ocular Fluid Staphylococcus aureus and S. epidermidis

**Figure 1:**
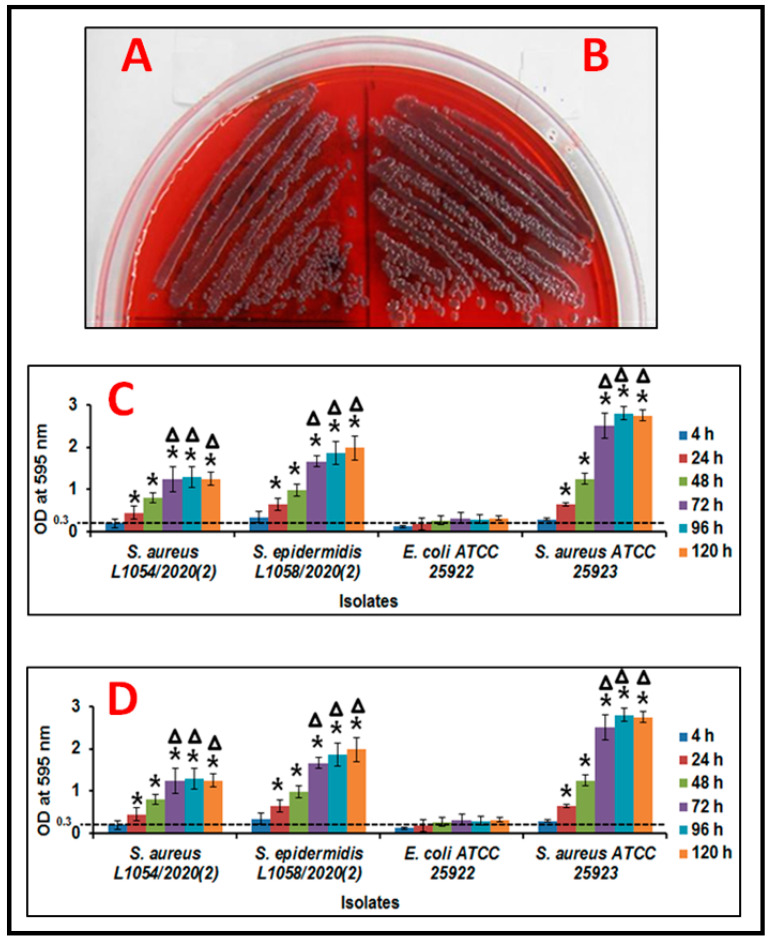
Biofilm formation in ocular fluid *Staphylococcus aureus* L-1054/2020(2) (**A**) and *S. epidermidis* L-1058/2020(2) (**B**) by the Congo red agar method (**A**,**B**), crystal violet method (**C**) and XTT method (**D**). *E. coli* ATCC 25922 was used as a negative control and *S. aureus* 25923 was used as a positive control. Each bar in (**C**,**D**) represent average value ± standard deviation. Experiments were performed in triplicates. Significance was calculated against 4 h biofilm using statistical analysis such as unpaired *t*-test and *p* value calculation. Experiments were performed in triplicates. * Indicates significant increase in biofilm formation compared to 4 h based on *t*-test and *p* value calculation. (*p* ≤ 0.05).

Ocular fluid *S. aureus* and *S. epidermidis* grew as black colonies on CRA plates indicative of biofilm formation (Figure 1A,B). Further, the Crystal Violet and XTT methods confirmed the biofilm formation potential of the two isolates (Figure 1C,D). In the CV method both *S. aureus* and *S. epidermidis* showed increase in biofilm formation from 4–48 h and reached a peak at 72 h (*p* ≤ 0.05) after which it stabilized (96 and 120 h). This was also in accordance with *S. aureus* ATCC 25923 the positive control for biofilm formation which also showed a similar trend in increase in biofilm formation. The negative control *E. coli* ATCC 25922 did not show any biofilm formation (Figure 1C). Interestingly, when biofilm formation was monitored by the XTT method it was observed that both *S. aureus* and *S. epidermidis* were probably more efficient in biofilm formation potential and by 48–72 h reached peak biofilm formation which was sustained till 120 h (Figure 1D). Experiments were performed in triplicates.

**Figure 2:**
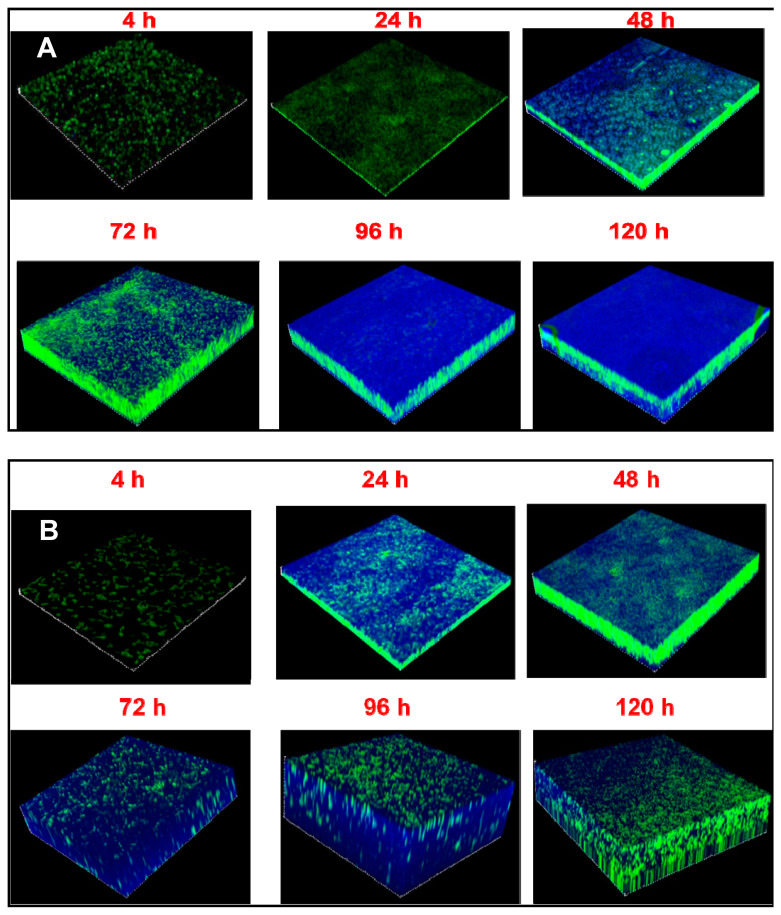
Temporal increase in thickness of biofilm in ocular *Staphylococcus aureus* L-1054/2020(2) (**A**) and *S. epidermidis* L-1058/2020(2) (**B**) by confocal scanning laser microscopy monitored between 4 to 120 h of biofilm growth on cover slips. The biofilm was stained with Syto9^®^ and Calcofluor white M2R. Viable cells appear green in color and EPS appears blue in color.

CLSM studies indicated that both in *S. aureus* and *S. epidermidis* the biofilm on cover slips stained positively between 4–120 h and EPS, which appeared blue in color, was clearly visible between 48–120 h (Figure 2A) in *S. aureus* but in *S. epidermidis* EPS was observed by 4 h and was visible even at 120 h (Figure 2B). CLSM studies also indicated that the thickness of biofilm of *S. aureus* on cover slips increased from 2 ± 0.25 to 11.96 ± 0.90 µm between 4 to 72 h of biofilm growth, after which between 72–120 h the thickness sustained between 11.96 ± 0.90 µm and 12.99 ± 0.46 µm at 120 h (Figure 2A and Table 1). In *S. epidermidis* also the thickness of the biofilm increased with time and continued to increase in thickness from 4–96 h after which there was slight decrease in thickness (Figure 2B and Table 1). Statistical analysis indicated that the thickness of the biofilm both in *S. aureus* and *S. epidermidis* was significantly increased after 48 h compared to the thickness at 4 h (Table 1).

### 3.2. Monitoring Biofilm Formation in Ocular Fluid Staphylococcus aureus and S. epidermidis by Scanning Electron Microscopy Using Cover Slips as a Substratum

**Figure 3:**
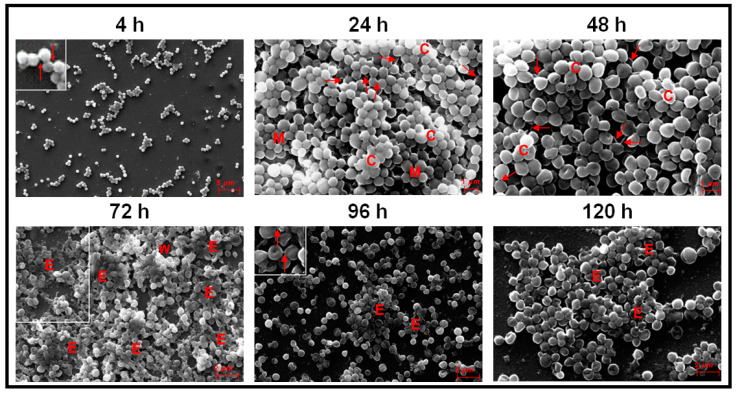
Scanning Electron Microscopy monitoring of biofilm formation in ocular surface *Staphylococcus aureus* L-1054/2020(2) between 4 to 120 h of biofilm growth on a cover slip. Single arrows represent intercellular connections (4–120 h), E represents EPS (72–120 h), C represents a column (24 and 48 h), M represents micro-colony (24 h) and W represents a water channel (72 h). Inset at 4 and 96 h represent intercellular connections and inset at 72 h represents EPS.

Ocular *S. aureus* at 4 h were attached to the cover slips as dispersed cells in groups of 2 or more or and mostly as small mono-layer of cells (Figure 3). At 24 h, a microcolony of multilayer clumping of cells was visible (>4 layers of cells) which also gets transformed into column-like structures between 24–48 h (Figure 3). Copious amount of EPS was formed between 72–120 h of biofilm formation (E in Figure 3). Intercellular connections were seen at 4, 24, 48 and 96 h (single arrows in Figure 3) but not clearly visible at 72 h, may be due to excessive of EPS at this stage. By 96 to 120 h, the clumps showed a tendency to disperse and several single cells were visible. The morphology of the cells was discernible between 4–120 h of biofilm formation. (Figure 3).

**Figure 4:**
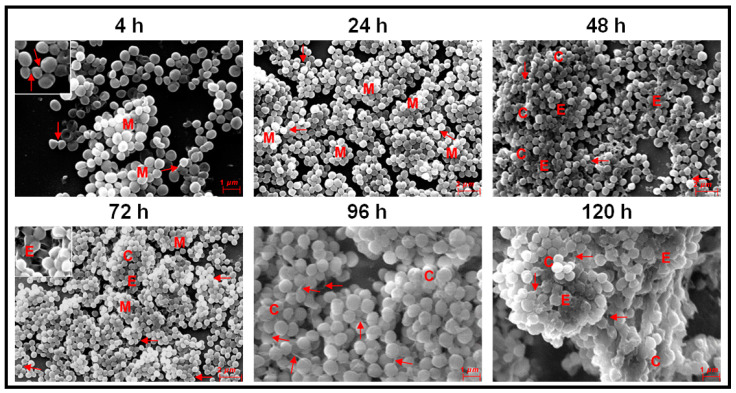
Scanning Electron Microscopy monitoring of biofilm formation in ocular *Staphylococcus epidermidis* L-1058/2020(2) between 4 to 120 h of biofilm growth on a cover slip. Single arrow represents intercellular connections (4–120 h), C represents a column (48–120 h), E represent EPS (48–120 h) and M represents microcolony (4–72 h).

In ocular fluid *S. epidermidis* also, attachment of cells to the substratum occurred at 4 h and small multilayer clumps which by 24 h were very prominent (Figure 4). Column-like structures were seen between 48–120 h (Figure 4) and EPS were clearly seen at 24–120 h of biofilm formation. Intercellular connections were visible at all stages of biofilm formation from 4–120 h (arrow) and were prominent at 96 h (Figure 4). Two adjacent prominent columns were visible at 120 h and the morphology of the cells in part of the column was obliterated (Figure 4).

### 3.3. Monitoring Biofilm Formation in Ocular Fluid Staphylococcus aureus and S. epidermidis by SEM Using Cornea as a Substratum

**Figure 5:**
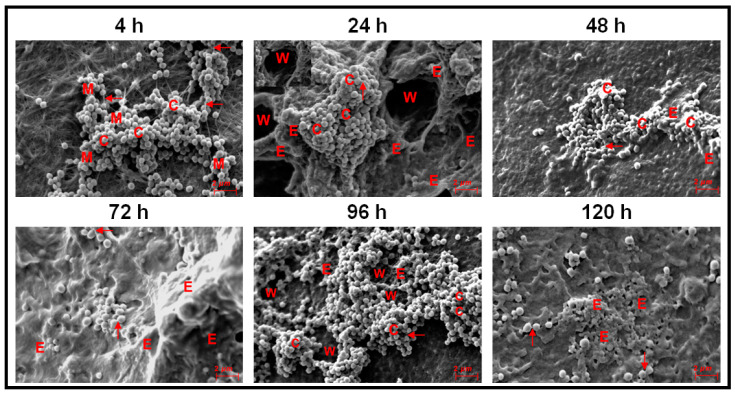
Scanning Electron Microscopy monitoring of biofilm formation in ocular *Staphylococcus aureus* L-1054/2020(2) between 4 to 120 h of biofilm growth on cadaveric cornea. Single arrow represents intercellular connections (4–48 h), C represent a column (4, 24, 48 and 96 h), E represents EPS (24–120 h), M represents microcolony (4 h) and W represents a water channel (24 and 96 h).

Biofilm formation by ocular *S. aureus* on cadaveric cornea was different from that observed on cover slips. By 4 h, cells adhered to the cornea, micro-colonies of multilayer cells and a small column were observed (Figure 5). Between 24–96 h, column-like structures were visible (Figure 5). By 48 h the column-like structures were less prominent and by 72 h, these structures were enclosed in EPS and not visible. By 96 h the columns reduced in size. A few single cells were visible by 48 h and the number of single cells increased by 120 h. The appearance of the single cells implied that the biofilm had entered the dispersal phase (Figure 5). In contrast to the biofilm on cover slip *S. aureus* also showed prominent water channels at 24 and 96 h of biofilm growth. Further, intercellular connections were seen between 4–120 h of biofilm formation (Figure 5).

**Figure 6:**
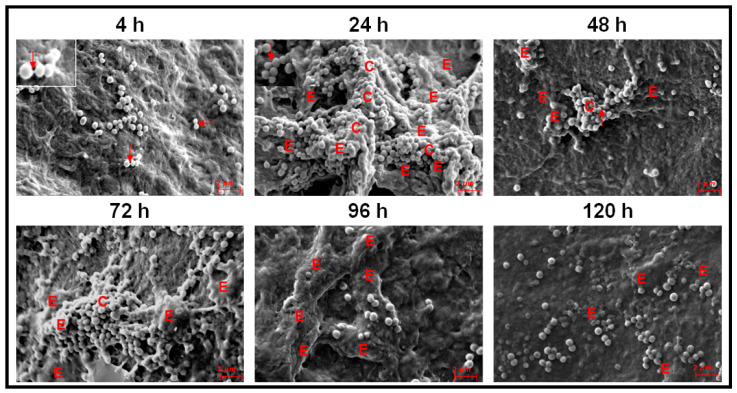
Scanning Electron Microscopy monitoring of biofilm formation in ocular surface *Staphylococcus epidermidis* L-1058/2020(2) between 4 to 120 h of biofilm growth on cadaveric cornea. Single arrow represents intercellular connections (4–48 h) (see inset at 4 h), C represents a column (24–72 h) and E represents EPS (24–120 h).

In ocular fluid *S. epidermidis* also attachment of cells and multilayer clumps of cells visible at 4 h and by 24 h column-like structures developed which were also visible at 72 h of biofilm formation (Figure 6). EPS was present in copious amounts between 24–120 h and intercellular connections were seen between 4 and 48 h of biofilm formation (Figure 6). The appearance of single cells between 48 to 120 h was indicative of the dispersal phase of the biofilm (Figure 6).

### 3.4. Antibiotic Susceptibility in Ocular Staphylococcus aureus and S. epidermidis in the Planktonic and Biofilm Phase

A total of 22 different antibiotics were evaluated for their MIC on the growth of ocular fluid *Staphylococcus aureus* and *S. epidermidis.* In the biofilm phase, the MIC increased several fold compared to the bacteria in the planktonic phase. In the biofilm phase *Staphylococcus aureus* showed 2.6 (monocycline) to 51.2 (gatifloxacin) fold increase in MIC whereas *S. epidermidis* showed 3.2 (vancomycin) to 85.3 (amikacin) fold increase in MIC depending on the antibiotic used (Table 2).

## 4. Discussion

This study confirms earlier observations demonstrating that ocular *Staphylococcus aureus* and *S. epidermidis* collected from the cornea, conjunctiva, eyelid margin, intraorbital foreign body, intraocular lenses, vitreous and aqueous humors of corneal ulcer patients and from patients with other ocular diseases including endophthalmitis exhibit biofilm-forming capacity as determined by CRA [54], CV [54,76], and SEM methods [55,77,78]. However, temporal dynamics of the biofilm formation by ocular *Staphylococcal* spp. from attachment to dispersal phase were rarely monitored [53,54,58]. Hou et al. [54] using SEM, observed that ocular biofilm-positive staphylococcal strains go through a phase of adhesion and accumulate as small monolayer sheets which then get transformed into multilayer sheets enclosed in the self-secreted EPS. By 24–72 h, biofilm structures (vertical columns or mushroom-like assemblies) were formed, and bacterial clusters were enclosed in the self-secreted EPS. Water channels and thread-like appendages between cells were also distinctly observed [54]. The present study on ocular fluid *Staphylococcus aureus* and *S. epidermidis* when grown on cover slips or cadaveric cornea demonstrated the potential of the two isolates to form biofilm on both abiotic and biotic substrata. When cover slips was used as the substratum both *S. aureus* and *S. epidermidis* go through an adhesion phase by 4 h. This phase is a key stage in the formation of biofilms and surface structures such as pili, fimbriae and flagella are very important in studies related to the dynamics of biofilm formation [48]. These motility organelles such as fimbriae [79], pili and flagella [80] and other surface proteins such as autolysin [81,82], exo-polysaccharides [83], extracellular DNA [84] and bacterial microbial surface components [85] interact with matrix proteins such as fibrinogen [86] and fibronectin [87] and facilitate adhesion of the cells to the substratum [88]. Castonguay et al. [89] demonstrated that a particular strain of *E. coli* PHL565 was unable to attach to solid surfaces and form a biofilm, but the strain of *E. coli* PHL565, in mixed cultures with *Pseudomonas putida* MT2 resulted in co-adhesion and in the formation of a mixed *E. coli* and *P. putida* biofilm, on glass surfaces. In contrast, *E. coli* with *Staphylococcus epidermidis* did not form a biofilm. It was suggested that mixed biofilms might represent an important mechanism, and a possible alternative strategy to form a biofilm when one of the partners does produce adhesion determinants. Such a strategy may help to increase the virulence of bacteria with low biofilm-forming potential. After the adhesion phase, in the proliferation phase, multi-layered colonies were formed between 24–48 h in *S. aureus* (Figure 3) and in *S. epidermidis* by 24 h (Figure 4). The maturation phase when the biofilm appears as vertical columns or mushroom-like assemblies enclosed in EPS was not very prominent in *S. aureus* (Figure 3, 24 h) but very well developed in *S. epidermidis* (Figure 4, 24–120 h). A feature that discriminated biofilm formation in ocular fluid *S. aureus* and *S. epidermidis* is that the former exhibited single cells between 96–120 h indicative of dispersion phase but in the same time frame *S. epidermidis* was still in the pen-ultimate maturation phase exhibiting a prominent column (Figure 3 and Figure 4). In the dispersal phase individual cells and/or multicellular aggregates are dispersed from the mature biofilms to seed new biofilms [20,41,42]. In contrast when biofilm formation of ocular fluid *S. aureus* and *S. epidermidis* were monitored on cadaveric cornea the dynamics of biofilm formation differed from that observed on cover slips. Both the isolates went through the 4 phases. Adhesion and microcolony formation were observed by 4 h, column-like structures were visible in both between 24 h to 96 h and the dispersion phase indicated by the appearance of single cells was visible by 48 h and beyond (Figure 5 and Figure 6). Thus biofilm formation appears to be more rapid on cadaveric cornea compared to when cover slip was used as the substratum. These studies imply that the temporal dynamics of biofilm formation was dependent on the substratum on which the biofilm was grown.

Earlier studies on biofilm formation in ocular isolates of *S. aureus* and *S. epidermidis* were done using synthetic material such as silicone, polymethyl-acrylate, hydrophilic acrylic, hydrophobic acrylic, etc. [25,62,63,90], to understand their biofilm formation ability on indwelling devices including contact lenses, sutures, scleral buckles, valvular tubes and keratoprostheses [20,24,25,26,52,77,91,92,93]. In our opinion, monitoring biofilm formation of ocular fluid *S. aureus* and *S. epidermidis* on cadaveric cornea is equally important because they are a major source of hospital-acquired infections and more importantly, corneal biofilms have been reported following experimental keratitis in mice [77], in patients with infectious crystalline keratopathy [94,95,96] or pterygium scleritis [97] and also in the absence of prosthetic material and in the absence of active corneal inflammation or infection [59]. Comparison of the biofilms formed on cover slips with that of cadaveric cornea indicated that EPS secretion was more copious on cornea (Figure 5 and Figure 6) than on cover slip (Figure 3 and Figure 4). Unknown host factors associated with the cadaveric cornea may facilitate copious EPS secretion. Generally bacterial cells bind to the host binding molecules, including fibrinogen, host extracellular matrix proteins, fibronectin and components of the blood plasma and such proteins have been detected as mediators of adhesion in clinical *Staphylococcal* isolates [98,99] which facilitate the binding of bacterial adhesins [100] to the surface.

A biofilm is also defined based on its architecture [29,30,32,33,34] which includes, in addition to the microbial cells and EPS, also some structural attributes such as the thickness (varying from monolayer of cells to structures such as columns and mushrooms) water channels [35] and the presence of voids. In this study it was observed that EPS was prominent in the biofilms of both the ocular fluid *S. aureus* and *S. epidermidis* and more so when the two were grown on cadaveric cornea. This observation is not surprising since EPS is an integral part of the biofilm architecture and is also known to maintain the structural integrity of the biofilm, anchor the biofilm to a substratum [44], facilitate cell to cell communication and the viability of cells by modulating substrate absorption, oxygen diffusion and transport of molecules within the biofilm [45,46]. In this study, thickness of the biofilm, yet another architectural feature was monitored by confocal microscopy as reported earlier [56,57]. The thickness of the biofilm ofocular fluid *S. aureus* and *S. epidermidis* increased with time and this is in accordance with the CV and XTT results which indicated increase in biofilm formation with time. However, based on the SEM results one should have observed a decrease in thickness by 120 h when the biofilm attains the dispersal phase. The observed discrepancy could be attributed to the fact that in the CV and XTT methods the substratum used was a polystyrene plate (Nunclon™, Thermo scientific, Roskilde, Denmark) which is different from the glass cover slip and definitely different from the cadaveric cornea. Earlier studies have indicated that biofilms release and disperse cells into the environment to colonize new sites [101]. This dispersal phase is a complex process and involves numerous environmental signals, signal transduction pathways and effectors [102]. That decrease in biomass is due to dispersal and not death of cells was elegantly demonstrated by Barraud et al. [103] who assessed biofilm dispersal as a concomitant decrease in biofilm biomass and an increase in planktonic biomass [103]. Other biofilm architectural features such as columns were also seen when ocular fluid *S. aureus* and *S. epidermidis* were grown either on cover slips or cadaveric cornea (Figure 3, Figure 4, Figure 5 and Figure 6) and water channels could also be identified only when grown on cadaveric cornea. Voids were not detected in the biofilms since voids are normally detected by time lapse monitoring of the biofilm after every 15 min [36,37,38].

Our studies also confirm that cells in the biofilm phase are several fold more resistant to antibiotics, a phenomenon associated with biofilm formation in bacteria [20,26,27,28,62,63]. Earlier studies had indicated that several ocular bacteria including *S. epidermidis, S. aureus* and *Streptococcus* spp. form biofilms [104] and majority of them were resistant to antibiotics. Further, our results were in accordance with these earlier studies which had demonstrated that the MIC of the antibiotic in the biofilm phase was significantly greater than that required for killing the cells in the planktonic phase [91,105]. This increase in MIC in the biofilm phase could be attributed to: inefficient penetration of the drug into the biofilm [106], inability of the drug to exerts its effect within the biofilm [107], transformation of the microorganisms in the biofilm into viable-but-nonculturable state [108], emergence of persister cells which are resistant to drugs [109], ability to survive under nutrient and oxygen limitation conditions and up-regulation of drug resistance-associated genes [105], ability of EPS to limit diffusion of aminoglycosides [110], ability of EPS to inactivate antibiotics [111], acquiring resistance to phagocytosis and induction of LPS modification genes [112]. A few of the strategies that have been demonstrated in ocular isolates as responsible for AMR include biofilm formation as indicated above and a high concordance between the presence of AMR genes and antibiotic resistance in 10 ocular *E. coli* strains and the presence of several virulent genes (*fimB* to *fimI*, *papB* to *papX*, etc.) and prophages (Enterobacteria phage HK97, Enterobacteria phage P1, *Escherichia *phage *D108*, etc.) which were unique to ocular *E. coli* [62,63,113]. To the best of our knowledge, this is probably the first study on biofilm forming potential of ocular *S. aureus* and *S. epidermidis* on cadaveric cornea from the attachment to the dispersal phase of biofilm formation.

## Figures and Tables

**Table 1:** Thickness of the biofilm (Z axis in µm) on cover slips in ocular *Staphylococcus aureus* and *S. epidermidis* as determined by confocal scanning laser microscopy.

Biofilm Formation (h)	*S. aureus* Biofilm Thickness (Z Axis, Average ± Standard Deviation in µm)	*S. epidermidis* Biofilm Thickness (Z Axis, Average ± Standard Deviation in µm)
4	2.00 ± 0.25	2.35 ± 0.19
24	2.36 ± 0.23 *	3.68 ± 0.26 *
48	4.68 ± 0.60 *	6.98 ± 0.45 *
72	11.96 ± 0.90 *	12.35 ± 0.16 *
96	12.06 ± 0.11 *	18.36 ± 0.21 *
120	12.99 ± 0.46 *	16.35 ± 0.16 *

* Experiments were performed in triplicate. For each sample, 10 randomly selected microscopic fields were chosen and the thickness was measured and the average and standard deviation for each time point was calculated. Significant change in thickness was calculated against the thickness at 4 h based on *t*-test and *p*-value calculation. Indicates significant increase in biofilm formation compared to 4 h (*p* ≤ 0.05).

**Table 2:** Antibiotic susceptibility of *Staphylococcus aureus* and *S. epidermidis* in the planktonic and biofilm phase.

Antibiotic	*S. aureus* MIC * (µg/mL)		*S. epidermidis* MIC * (µg/mL)		Fold Change ** in MIC (Planktonic vs. Biofilm Phase)	
	Planktonic Phase	Biofilm Phase	Planktonic Phase	Biofilm Phase	*S. aureus*	*S. epidermidis*
Amikacin	12	120	12	1024	10	85.3
Gentamicin	24	480	24	1024	20	42.7
Tobramycin	24	128	48	256	5.3	5.3
Ampicillin	24	256	48	1024	10.7	21.3
Cefuroxime	24	512	24	512	21.3	21.3
Ceftriaxone	12	512	12	512	42.7	42.7
Cefepime	48	1024	48	1024	21.3	21.3
Cefazolin	24	480	12	480	20	40
Ceftazidime	24	1024	24	1024	42.7	42.7
Gatifloxacin	20	1024	20	1024	51.2	51.2
Moxifloxacin	48	1024	48	1024	21.3	21.3
Ciprofloxacin	24	64	24	128	2.7	5.3
Ofloxacin	12	512	32	1024	42.7	32
Vancomycin	6	64	10	32	10.7	3.2
Chloramphenicol	12	32	20	128	2.7	6.4
Azithromycin	48	>1054	128	>1024	22	22
Metronidazole	24	>1054	24	>1024	22	22
Triamcinolone	12	128	24	128	10.6	5.3
Deriphyllin	6	64	6	256	10.6	42.6
Clindamycin	48	>1054	48	>1024	22	22
Lincomycin	24	512	32	1024	21.3	32
Monocycline	24	64	20	256	2.6	12.8

* MIC is the minimum concentration of the antibiotic required to inhibit biofilm formation completely. ** Fold change was measured by dividing the MIC in biofilm phase with the MIC in the planktonic phase.

## References

[1] McClellan K.A. (1997). Mucosal defense of the outer eye. Surv. Ophthalmol..

[2] McDermott A.M. (2013). Antimicrobial compounds in tears. Exp. Eye Res..

[3] Pleyer U., Baatz H. (1997). Antibacterial Protection of the Ocular Surface. Ophthalmologica.

[4] Shovlin J.P., Argüeso P., Carnt N., Chalmers R.L., Efron N., Fleiszig S.M.J., Nichols J.J., Polse K.A., Stapleton F., Wiley L. (2013). Ocular surface health with contact lens wear. Cont. Lens Anterior Eye.

[5] Chandler J.W., Cummings M., Gillette T.E. (1985). Presence of Langerhans cells in the central corneas of normal human infants. Investig. Ophthalmol. Vis. Sci..

[6] Gillette T.E., Chandler J.W., Greiner J.V. (1982). Langerhans Cells of the Ocular Surface. Ophthalmology.

[7] Akpek E.K., Gottsch J.D. (2003). Immune defense at the ocular surface. Eye.

[8] Dong Q., Brulc J.M., Iovieno A., Bates B., Garoutte A., Miller D., Revanna K.V., Gao X., Antonopoulos D.A., Slepak V.Z. (2011). Diversity of Bacteria at Healthy Human Conjunctiva. Investig. Ophthalmol. Vis. Sci..

[9] Graham J.E., Moore J.E., Jiru X., Moore J.E., Goodall E.A., Dooley J.S., Hayes V.E., Dartt D.A., Downes C.S., Moore T.C. (2007). Ocular pathogen or commensal: A PCR-based study of surface bacterial flora in normal and dry eyes. Investig. Ophthalmol. Vis. Sci..

[10] Ozkan J., Nielsen S., Diez-Vives C., Coroneo M., Thomas T., Willcox M. (2017). Temporal Stability and Composition of the Ocular Surface Microbiome. Sci. Rep..

[11] Schabereiter-Gurtner C., Maca S., Rolleke S., Nigl K., Lukas J., Hirschl A., Lubitz W., Barisani-Asenbauer T. (2001). 16S rDNA-based identification of bacteria from conjunctival swabs by PCR and DGGE fingerprinting. Investig. Ophthalmol. Vis. Sci..

[12] Shivaji S., Jayasudha R., Chakravarthy S.K., SaiAbhilash C.R., Sai Prashanthi G., Sharma S., Garg P., Murthy S.I. (2021). Alterations in the conjunctival surface bacterial microbiome in bacterial keratitis patients. Exp. Eye Res..

[13] Shivaji S., Jayasudha R., Sai Prashanthi G., Kalyana Chakravarthy S., Sharma S. (2019). The Human Ocular Surface Fungal Microbiome. Investig. Ophthalmol. Vis. Sci..

[14] Keilty R.A. (1930). The Bacterial Flora of the Normal Conjunctiva with Comparative Nasal Culture Study. Am. J. Ophthalmol..

[15] Bispo P.J.M., Melo G.B.D., d’Azevedo P.A., Höfling-Lima A.L., Yu M.C.Z., Pignatari A.C.C. (2008). Coagulase-Negative Staphylococcus (CoNS), *Staphylococcus aureus*, *S. epidermidis*, and *Streptococcus viridians* cause acute postoperative endophthalmitis. Arq. Bras. Ophthalmol..

[16] Han D.P., Wisniewski S.R., Wilson L.A., Barza M., Vine A.K., Doft B.H., Kelsey S.F. (1996). Spectrum and susceptibilities of microbiologic isolates in the Endophthalmitis Vitrectomy Study. Am. J. Ophthalmol..

[17] Kresloff M.S., Castellarin A.A., Zarbin M.A. (1998). Endophthalmitis. Surv. Ophthalmol..

[18] Melo G.B., Bispo P.J.M., Yu M.C.Z., Pignatari A.C.C., Höfling-Lima A.L. (2011). Microbial profile and antibiotic susceptibility of culture-positive bacterial endophthalmitis. Eye.

[19] Miller D.M., Vedula A.S., Flynn H.W., Miller D., Scott I.U., Smiddy W.E., Murray T.G., Venkatraman A.S. (2007). Endophthalmitis caused by *Staphylococcus epidermidis*: In vitro antibiotic susceptibilities and clinical outcomes. Ophthalmic Surg. Lasers Imaging.

[20] Behlau I., Gilmore M.S. (2008). Microbial biofilms in ophthalmology and infectious disease. Arch. Ophthalmol..

[21] Bispo P.J., Haas W., Gilmore M.S. (2015). Biofilms in infections of the eye. Pathogens.

[22] Catalanotti P., Lanza M., Del Prete A., Lucido M., Catania M.R., Galle F., Boggia D., Perfetto B., Rossano F. (2005). Slime-Producing *Staphylococcus epidermidis* and *S. aureus* in acute bacterial conjunctivitis in soft contact lens wearers. New Microbiol..

[23] Duggirala A., Kenchappa P., Sharma S., Peeters J.K., Ahmed N., Garg P., Das T., Hasnain S.E. (2007). High-Resolution genome profiling differentiated *Staphylococcus epidermidis* isolated from patients with ocular infections and normal individuals. Investig. Ophthalmol. Vis. Sci..

[24] Darouiche R.O. (2004). Treatment of infections associated with surgical implants. N. Engl. J. Med..

[25] Moreno A., dos Santos D.M., Lamartine de Moraes Melo Neto C., Luiz de Melo Moreno A., de Magalhães Bertoz A.P., Goiato M.C. (2020). In Vitro evaluation of the effect of different disinfectants on the biofilm of *Staphylococcus epidermidis* and *Staphylococcus aureus* formed on acrylic ocular prostheses. PLoS ONE.

[26] Paharik A.E., Horswill A.R. (2016). The Staphylococcal Biofilm: Adhesins, Regulation, and Host Response. Microbiol. Spectr..

[27] Fridkin S.K., Hageman J.C., Morrison M., Sanza L.T., Como-Sabetti K., Jernigan J.A., Harriman K., Harrison L.H., Lynfield R., Farley M.M. (2005). Methicillin-resistant *Staphylococcus aureus* disease in three communities. N. Engl. J. Med..

[28] Weigel L.M., Clewell D.B., Gill S.R., Clark N.C., McDougal L.K., Flannagan S.E., Kolonay J.F., Shetty J., Killgore G.E., Tenover F.C. (2003). Genetic Analysis of a High-Level Vancomycin-Resistant Isolate of *Staphylococcus aureus*. Science.

[29] Costerton J.W., Cheng K.J., Geesey G.G., Ladd T.I., Nickel J.C., Dasgupta M., Marrie T.J. (1987). Bacterial Biofilms in Nature and Disease. Annu. Rev. Microbiol..

[30] Costerton J.W., Lewandowski Z., Caldwell D.E., Korber D.R., Lappin-Scott H.M. (1995). Microbial Biofilms. Annu. Rev. Microbiol..

[31] Nikolaev Y.A., Plakunov V.K. (2007). Biofilm—“City of microbes” or an analogue of multicellular organisms?. Microbiology.

[32] Donlan R.M. (2000). Role of Biofilms in Antimicrobial Resistance. ASAIO J..

[33] Tolker-Nielsen T., Brinch U.C., Ragas P.C., Andersen J.B., Jacobsen C.S., Molin S. (2000). Development and Dynamics of *Pseudomonas* sp. Biofilms. J. Bacteriol..

[34] Tolker-Nielsen T., Molin S. (2000). Spatial Organization of Microbial Biofilm Communities. Microb. Ecol..

[35] Lewandowski Z. (2000). Structure and function of biofilms. Evans LV, e. Biofilms: Recent Advances in Their Study and Control.

[36] Tombolini R., Unge A., Davey M.E., de Bruijn F.J., Jansson J.K. (1997). Flow cytometric and microscopic analysis of GFP-tagged *Pseudomonas fluorescens* bacteria. FEMS Microbiol. Ecol..

[37] Andersen J.B., Sternberg C., Poulsen L.K., Bjørn S.P., Givskov M., Molin S. (1998). New Unstable Variants of Green Fluorescent Protein for Studies of Transient Gene Expression in Bacteria. Appl. Environ. Microbiol..

[38] Yarwood J.M., Bartels D.J., Volper E.M., Greenberg E.P. (2004). Quorum Sensing in *Staphylococcus aureus* Biofilms. J. Bacteriol..

[39] Davies D. (2003). Understanding biofilm resistance to antibacterial agents. Nat. Rev. Drug Discov..

[40] O’Toole G.A., Kolter R. (1998). Initiation of biofilm formation in *Pseudomonas fluorescens* WCS365 proceeds via multiple, convergent signalling pathways: A genetic analysis. Mol. Microbiol..

[41] Guilhen C., Forestier C., Balestrino D. (2017). Biofilm dispersal: Multiple elaborate strategies for dissemination of bacteria with unique properties. Mol. Microbiol..

[42] Joo H.-S., Otto M. (2012). Molecular Basis of In Vivo Biofilm Formation by Bacterial Pathogens. Chem. Biol..

[43] Pan M., Zhu L., Chen L., Qiu Y., Wang J. (2010). Detection Techniques for Extracellular Polymeric Substances in Biofilms: A Review. BioResources.

[44] Characklis W.G., Marshall K.C., Characklis W.G., Marshall K.C. (1990). Biofilms: A Basis for an Interdisciplinary Approach.

[45] Branda S.S., Vik Å., Friedman L., Kolter R. (2005). Biofilms: The matrix revisited. Trends Microbiol..

[46] Flemming H.-C., Wingender J. (2010). The biofilm matrix. Nat. Rev. Microbiol..

[47] McBain A.J. (2009). In Vitro Biofilm Models: An Overview. Adv. Appl. Microbiol..

[48] Burtseva O., Baulina O., Zaytseva A., Fedorenko T., Chekanov K., Lobakova E. (2021). In vitro Biofilm Formation by Bioluminescent Bacteria Isolated from the Marine Fish Gut. Microb. Ecol..

[49] Silva V.O., Soares L.O., Silva Júnior A., Mantovani H.C., Chang Y.-F., Moreira M.A.S. (2014). Biofilm Formation on Biotic and Abiotic Surfaces in the Presence of Antimicrobials by *Escherichia coli* Isolates from Cases of Bovine Mastitis. Appl. Environ. Microbiol..

[50] Domínguez-Manzano J., León-Romero Á., Olmo-Ruiz C., Bautista-Gallego J., Arroyo-López F.N., Garrido-Fernández A., Jiménez-Díaz R. (2012). Biofilm formation on abiotic and biotic surfaces during Spanish style green table olive fermentation. Int. J. Food Microbiol..

[51] Guiotti A.M., da Silva E.V.F., Catanoze I.A., de Carvalho K.H.T., Malavazi E.M., Goiato M.C., Dos Santos D.M., de Almeida M.T.G. (2018). Microbiological analysis of conjunctival secretion in anophthalmic cavity, contralateral eye and ocular prosthesis of patients with maxillofacial abnormalities. Lett. Appl. Microbiol..

[52] Paranhos R.M.Z.F., Batalhão C.H., Semprini M., Regalo S.C.H., Ito I.Y., Mattos M.D.G.C.D. (2007). Evaluation of ocular prosthesis biofilm and anophthalmic cavity contamination after use of three cleansing solutions. J. Appl. Oral Sci..

[53] Kirchhoff L., Arweiler-Harbeck D., Arnolds J., Hussain T., Hansen S., Bertram R., Buer J., Lang S., Steinmann J., Höing B. (2020). Imaging studies of bacterial biofilms on cochlear implants—Bioactive glass (BAG) inhibits mature biofilm. PLoS ONE.

[54] Hou W., Sun X., Wang Z., Zhang Y. (2012). Biofilm-Forming capacity of *Staphylococcus epidermidis*, *Staphylococcus aureus*, and *Pseudomonas aeruginosa* from ocular infections. Investig. Ophthalmol. Vis. Sci..

[55] Cerca N., Martins S., Cerca F., Jefferson K.K., Pier G.B., Oliveira R., Azeredo J. (2005). Comparative assessment of antibiotic susceptibility of coagulase-negative staphylococci in biofilm versus planktonic culture as assessed by bacterial enumeration or rapid XTT colorimetry. J. Antimicrob. Chemother..

[56] Baidamshina D.R., Trizna E.Y., Holyavka M.G., Bogachev M.I., Artyukhov V.G., Akhatova F.S., Rozhina E.V., Fakhrullin R.F., Kayumov A.R. (2017). Targeting microbial biofilms using Ficin, a nonspecific plant protease. Sci. Rep..

[57] Jefferson K.K., Goldmann D.A., Pier G.B. (2005). Use of Confocal Microscopy to Analyze the Rate of Vancomycin Penetration through *Staphylococcus aureus* Biofilms. Antimicrob. Chemother..

[58] Sonesson A., Przybyszewska K., Eriksson S., Mörgelin M., Kjellström S., Davies J., Potempa J., Schmidtchen A. (2017). Identification of bacterial biofilm and the *Staphylococcus aureus* derived protease, staphopain, on the skin surface of patients with atopic dermatitis. Sci. Rep..

[59] Cordoba A., Graue-Hernandez E.O., Bermudez-Magner J.A., Ramirez-Miranda A., Irusteta L., Bautista-de Lucio V.M., Ponce-Angulo D.G., Bautista-Hernandez L.A., Navas A. (2019). Corneal Biofilm Plaques: A Novel Clinical Presentation. Cornea.

[60] Nonhoff C., Rottiers S., Struelens M.J. (2005). Evaluation of the Vitek 2 system for identification and antimicrobial susceptibility testing of *Staphylococcus* spp.. Clin. Microbiol. Infect..

[61] Smith J.L., Dell B.J. (1990). Capability of Selective Media to Detect Heat-Injured Shigella flexneri. J. Food Prot..

[62] Ranjith K., Arunasri K., Reddy G.S., Adicherla H., Sharma S., Shivaji S. (2017). Global gene expression in *Escherichia coli*, isolated from the diseased ocular surface of the human eye with a potential to form biofilm. Gut Pathog..

[63] Ranjith K., Ramchiary J., Prakash J.S.S., Arunasri K., Sharma S., Shivaji S. (2019). Gene Targets in Ocular Pathogenic *Escherichia coli* for Mitigation of Biofilm Formation to Overcome Antibiotic Resistance. Front. Microbiol..

[64] Freeman D.J., Falkiner F.R., Keane C.T. (1989). New method for detecting slime production by coagulase negative staphylococci. J. Clin. Pathol..

[65] Romero D., Aguilar C., Losick R., Kolter R. (2010). Amyloid Fibers Provide Structural Integrity to *Bacillus subtilis* Biofilms. Proc. Natl. Acad. Sci. USA.

[66] Reichhardt C., Jacobson A.N., Maher M.C., Uang J., McCrate O.A., Eckart M., Cegelski L. (2015). Congo Red Interactions with Curli-Producing *E. coli* and Native Curli Amyloid Fibers. PLoS ONE.

[67] Phuengmaung P., Somparn P., Panpetch W., Singkham-In U., Wannigama D.L., Chatsuwan T., Leelahavanichkul A. (2020). Coexistence of *Pseudomonas aeruginosa* With Candida albicans Enhances Biofilm Thickness Through Alginate-Related Extracellular Matrix but Is Attenuated by N-acetyl-l-cysteine. Front. Cell Infect. Microbiol..

[68] Cruz C.D., Shah S., Tammela P. (2018). Defining conditions for biofilm inhibition and eradication assays for Gram-positive clinical reference strains. BMC Microbiol..

[69] Dhale R.P., Ghorpade M.V., Dharmadhikari C.A. (2014). Comparison of various methods used to detect biofilm production of *Candida* species. J. Clin. Diagn. Res..

[70] Sabaeifard P., Abdi-Ali A., Soudi M.R., Dinarvand R. (2014). Optimization of tetrazolium salt assay for *Pseudomonas aeruginosa* biofilm using microtiter plate method. J. Microbiol. Methods.

[71] Cowan S.E., Gilbert E., Liepmann D., Keasling J.D. (2000). Commensal Interactions in a Dual-Species Biofilm Exposed to Mixed Organic Compounds. Appl. Environ. Microbiol..

[72] Ledeboer N.A., Jones B.D. (2005). Exopolysaccharide Sugars Contribute to Biofilm Formation by *Salmonella enterica serovar Typhimurium* on HEp-2 Cells and Chicken Intestinal Epithelium. J. Bacteriol..

[73] Aquavella J.V., Van Horn D.L., Haggerty C.J. (1975). Corneal Preservation Using M-K Medium. Am. J. Ophthalmol..

[74] Pinnock A., Shivshetty N., Roy S., Rimmer S., Douglas I., MacNeil S., Garg P. (2017). Ex Vivo rabbit and human corneas as models for bacterial and fungal keratitis. Graefes Arch. Clin. Exp. Ophthalmol..

[75] CLSI (2012). Performance Standards for Antimicrobial Susceptibility Testing.

[76] Okajima Y., Kobayakawa S., Tsuji A., Tochikubo T. (2006). Biofilm formation by *Staphylococcus epidermidis* on intraocular lens material. Investig. Ophthalmol. Vis. Sci..

[77] Saraswathi P., Beuerman R.W. (2015). Corneal Biofilms: From Planktonic to Microcolony Formation in an Experimental Keratitis Infection with *Pseudomonas aeruginosa*. Ocul. Surf..

[78] Xu Z., Liang Y., Lin S., Chen D., Li B., Li L., Deng Y. (2016). Crystal Violet and XTT Assays on *Staphylococcus aureus* Biofilm Quantification. Curr. Microbiol..

[79] Blumer C., Kleefeld A., Lehnen D., Heintz M., Dobrindt U., Nagy G., Michaelis K., Emödy L., Polen T., Rachel R. (2005). Regulation of type 1 fimbriae synthesis and biofilm formation by the transcriptional regulator LrhA of *Escherichia coli*. Microbiology.

[80] Déziel E., Comeau Y., Villemur R. (2001). Initiation of Biofilm Formation by *Pseudomonas aeruginosa* 57RP Correlates with Emergence of Hyperpiliated and Highly Adherent Phenotypic Variants Deficient in Swimming, Swarming, and Twitching Motilities. J. Bacteriol..

[81] Büttner H., Mack D., Rohde H. (2015). Structural basis of *Staphylococcus epidermidis* biofilm formation: Mechanisms and molecular interactions. Front. Cell. Infect. Microbiol..

[82] Gross M., Cramton S.E., Götz F., Peschel A. (2001). Key Role of Teichoic Acid Net Charge in *Staphylococcus aureus* Colonization of Artificial Surfaces. Infect. Immun..

[83] Ma L., Conover M., Lu H., Parsek M.R., Bayles K., Wozniak D.J. (2009). Assembly and Development of the *Pseudomonas aeruginosa* Biofilm Matrix. PLoS Pathog..

[84] Pakkulnan R., Anutrakunchai C., Kanthawong S., Taweechaisupapong S., Chareonsudjai P., Chareonsudjai S. (2019). Extracellular DNA facilitates bacterial adhesion during *Burkholderia pseudomallei* biofilm formation. PLoS ONE.

[85] Clarke S.R., Foster S.J. (2006). Surface Adhesins of *Staphylococcus aureus*. Adv. Microb. Physiol..

[86] Pei L., Palma M., Nilsson M., Guss B., Flock J.-I. (1999). Functional Studies of a Fibrinogen Binding Protein from *Staphylococcus epidermidis*. Infect. Immun..

[87] McElroy M.C., Cain D.J., Tyrrell C., Foster T.J., Haslett C. (2002). Increased Virulence of a Fibronectin-Binding Protein Mutant of *Staphylococcus aureus* in a Rat Model of Pneumonia. Infect. Immun..

[88] Li M., Du X., Villaruz A.E., Diep B.A., Wang D., Song Y., Tian Y., Hu J., Yu F., Lu Y. (2012). MRSA epidemic linked to a quickly spreading colonization and virulence determinant. Nat. Med..

[89] Castonguay M.-H., van der Schaaf S., Koester W., Krooneman J., van der Meer W., Harmsen H., Landini P. (2006). Biofilm formation by *Escherichia coli* is stimulated by synergistic interactions and co-adhesion mechanisms with adherence-proficient bacteria. Res. Microbiol..

[90] Baillif S., LeDuff F., Hartmann D.J., Kodjikian L. (2013). *Staphylococcus epidermidis* Biofilm Formation and Structural Organization on Different Types of Intraocular Lenses under in vitro Flow Conditions. Ophthalmic Res..

[91] Jassim S.H., Sivaraman K.R., Jimenez J.C., Jaboori A.H., Federle M.J., de la Cruz J., Cortina M.S. (2015). Bacteria Colonizing the Ocular Surface in Eyes with Boston Type 1 Keratoprosthesis: Analysis of Biofilm-Forming Capability and Vancomycin Tolerance. Investig. Ophthalmol. Vis. Sci..

[92] Otto M., Romeo T. (2008). Staphylococcal Biofilms. Bacterial Biofilms.

[93] Sivaraman K.R., Hou J.H., Chang J.H., Behlau I., Cortina M.S., De La Cruz J. (2016). Scanning Electron Microscopic Analysis of Biofilm Formation in Explanted Human Boston Type I Keratoprostheses. Cornea.

[94] Elder M.J., Matheson M., Stapleton F., Dart J.K.G. (1996). Biofilm Formation in Infectious Crystalline Keratopathy Due to *Candida albicans*. Cornea.

[95] Fulcher T.P., Dart J.K.G., McLaughlin-Borlace L., Howes R., Matheson M., Cree I. (2001). Demonstration of biofilm in infectious crystalline keratopathy using ruthenium red and electron microscopy. Ophthalmology.

[96] Georgiou T., Qureshi S.H., Chakrabarty A., Noble B.A. (2002). Biofilm formation and coccal organisms in infectious crystalline keratopathy. Eye.

[97] Mihara E., Shimizu M., Touge C., Inoue Y. (2004). Case of a large, movable bacterial concretion with biofilm formation on the ocular surface. Cornea.

[98] Herrmann M., Vaudaux P.E., Pittet D., Auckenthaler R., Lew P.D., Perdreau F.S., Peters G., Waldvogel F.A. (1988). Fibronectin, Fibrinogen, and Laminin Act as Mediators of Adherence of Clinical Staphylococcal Isolates to Foreign Material. J. Infect. Dis..

[99] McGavin M.H., Krajewska-Pietrasik D., Ryden C., Hook M. (1993). Identification of a *Staphylococcus aureus* extracellular matrix-binding protein with broad specificity. Infect. Immun..

[100] MacKintosh E.E., Patel J.D., Marchant R.E., Anderson J.M. (2006). Effects of biomaterial surface chemistry on the adhesion and biofilm formation of *Staphylococcus epidermidis* in vitro. J. Biomed. Mater. Res..

[101] Kaplan J.B. (2010). Biofilm Dispersal: Mechanisms, Clinical Implications, and Potential Therapeutic Uses. J. Dent. Res..

[102] Karatan E., Watnick P. (2009). Signals, Regulatory Networks, and Materials That Build and Break Bacterial Biofilms. Microbiol. Mol. Biol. Rev..

[103] Barraud N., Moscoso J.A., Ghigo J.-M., Filloux A., Filloux A., Ramos J.-L. (2014). Methods for Studying Biofilm Dispersal in *Pseudomonas aeruginosa*. Pseudomonas Methods and Protocols.

[104] Zegans M.E., Becker H.I., Budzik J., O’Toole G. (2002). The Role of Bacterial Biofilms in Ocular Infections. DNA Cell Biol..

[105] Kobayakawa S., Jett B.D., Gilmore M.S. (2005). Biofilm Formation by *Enterococcus faecalis* on Intraocular Lens Material. Curr. Eye Res..

[106] Mah T.-F.C., O’Toole G.A. (2001). Mechanisms of biofilm resistance to antimicrobial agents. Trends Microbiol..

[107] Xu K.D., McFeters G.A., Stewart P.S. (2000). Biofilm resistance to antimicrobial agents. Microbiology.

[108] Li L., Mendis N., Trigui H., Oliver J.D., Faucher S.P. (2014). The importance of the viable but non-culturable state in human bacterial pathogens. Front. Microbiol..

[109] Gerdes K., Maisonneuve E. (2012). Bacterial Persistence and Toxin-Antitoxin Loci. Annu. Rev. Microbiol..

[110] Khan W., Bernier S.P., Kuchma S.L., Hammond J.H., Hasan F., O’Toole G.A. (2010). Aminoglycoside resistance of *Pseudomonas aeruginosa* biofilms modulated by extracellular polysaccharide. Int. Microbiol..

[111] Billings N., Birjiniuk A., Samad T.S., Doyle P.S., Ribbeck K. (2015). Material properties of biofilms—A review of methods for understanding permeability and mechanics. Rep. Prog. Phys..

[112] Wilton M., Charron-Mazenod L., Moore R., Lewenza S. (2016). Extracellular DNA Acidifies Biofilms and Induces Aminoglycoside Resistance in *Pseudomonas aeruginosa*. Antimicrob. Agents Chemother..

[113] Ranjith K., SaiAbhilash C.R., Sai Prashanthi G., Padakandla S.R., Sharma S., Shivaji S. (2020). Phylogenetic Grouping of Human Ocular *Escherichia coli* Based on Whole-Genome Sequence Analysis. Microorganisms.

